# English as a Foreign Language Teacher Flow: How Do Personality and Emotional Intelligence Factor in?

**DOI:** 10.3389/fpsyg.2022.793955

**Published:** 2022-06-20

**Authors:** Alireza Sobhanmanesh

**Affiliations:** Faculty of Humanities and Social Sciences, Sheridan College, Oakville, ON, Canada

**Keywords:** the flow state, EFL teacher flow, the big five personality traits, conscientiousness and openness to experience, emotional intelligence

## Abstract

Teaching is one of the professions that creates opportunities for individuals to experience flow, a state of complete absorption in an activity. However, very few studies have examined ESL/EFL teachers’ flow states inside or outside the classroom. As such, this study aimed to explore the quality of experience of 75 EFL teachers in flow and also examine the relationships between their emotional intelligence, the Big Five personality traits and the flow state. To this end, the teachers filled out recurrent flow surveys for a week, and also completed emotional intelligence and the Big Five personality questionnaires. It was found that reading was the major flow trigger outside the classroom and teaching and delivering lessons was the most significant flow-inducing activity for the teachers inside the classroom. Furthermore, correlations and independent samples *t*-tests indicated that all emotional intelligence and personality traits had significant relationships with flow except agreeableness. Finally, multiple regression analysis showed that two personality traits, conscientiousness and openness to experience were the strongest predictors of the flow state. The implications for future flow-related research in the field of applied linguistics are discussed.

## Introduction

Recently, increasing attention has been paid to the issue of teacher motivation as an important way of improving the effectiveness of teaching and learning in the English as a Foreign Language (EFL) classroom (Dörnyei and Kubanyiova, 2014; Mercer and Kostoulas, 2018). There is now a growing recognition in the field of applied linguistics that learners’ well-being is closely connected to that of their teachers (Mercer and Kostoulas, 2018; Mercer and Gregersen, 2020). Through emotional contagion, a happy and motivated teacher can keep the learners highly motivated and happy which positively impacts on their classroom learning experiences. According to Csikszentmihalyi (1997), teachers who continuously enjoy learning about the subject matter and find the act of teaching intrinsically rewarding will be more likely to engage their learners and enhance their intrinsic motivation. As such, by being immersed in the act of teaching, teachers can demonstrate that learning can be a rewarding experience in and of itself and can, therefore, keep the learners engaged.

The term *flow*, as coined by Csikszentmihalyi (1975, 1990), accounts for moments of mastery and enjoyment. Flow is defined as the positive state of complete absorption and enjoyment in an activity (Csikszentmihalyi, 1990). Studies in applied linguistics show that the flow state can occur in the ESL classroom (Egbert, 2003; Czimmermann and Piniel, 2016). Based on flow theory (Csikszentmihalyi, 1975, 1990), it can be hypothesized that *teacher flow* occurs when a teacher is completely absorbed in the act of teaching. Their attention is fully invested in the task at hand, as they lose their sense of self-awareness. This happens when feedback is ample in the classroom environment, giving the teacher the opportunity to adjust the level of teaching to that of the learners, which subsequently leads to adjustments made on the part of learners to meet the new level of challenge (Csikszentmihalyi, 1975, 1990). This continual adaptation of the challenge level based on the learners’ level of skills enables the class group to continue experiencing flow and, in this way, provides them with the opportunity for continued growth and development (Csikszentmihalyi, 1975, 1990).

According to flow theory, certain personality types, i.e., *the autotelic personality*, are in flow more often and experience higher intensity flow compared to others (Nakamura and Csikszentmihalyi, 2002; Asakawa, 2004; Tse et al., 2020a). Individuals with autotelic tendencies undertake tasks for the intrinsic rewards they find in them, not for any external rewards (Csikszentmihalyi, 1990, 1997). Research in the field of psychology has shown that these individuals are able to create challenges for themselves in circumstances where others might get bored (Asakawa, 2004, 2010). They are also characterized by their curiosity and persistence and as a result have a higher sense of well-being (Tse et al., 2020b).

Although no studies have been done on autotelic ESL/EFL teachers to date, it can be hypothesized that these individuals play a fundamental role in the engagement of their students in the classroom, thus, bringing value to their institutions. Language teaching is characterized by high emotionality, motivational changes, shifting identities, and personally and culturally meaningful topics and material (MacIntyre et al., 2019), which differentiate it from the teaching of content (Borg, 2006). These characteristics can facilitate or impede the flow state in significant ways which makes it important to study EFL teacher flow. To this aim, this study seeks to understand the behavioral patterns which characterize autotelic EFL teachers and, more specifically, examine the relationship between flow and the Big Five personality traits (McCrae and Costa, 2008). Furthermore, as qualities such as emotional regulation and emotional management have been found instrumental to the flow experience (Marin and Bhattacharya, 2013; Freire and Tavares, 2016; Tavares and Freire, 2016; Xie, 2021), this study aims to investigate whether teachers who experience flow more often measure more positively on an emotional intelligence test, i.e., Bar-On’s EQ-i Inventory (Bar-On, 1997, 2004). Increased understanding of these characteristics and constructs might help language teachers enhance their flow proneness, i.e., the tendency to experience flow, which can cross over to their learners (Bakker, 2005) and create an optimal classroom environment characterized by intrinsic motivation (Hektner and Csikszentmihalyi, 1996), academic achievement (Joo et al., 2015), and self-esteem (Mao et al., 2020) among other factors.

## Flow Theory

In the early interviews that Csikszentmihalyi (1975, 1990) conducted with rock climbers, musicians and chess players, six dimensions were found to characterize the flow state: the merging of action and awareness, loss of self-consciousness, complete concentration on the task at hand, heightened sense of control, distortion of time, and the autotelic experience. When an individual is in the flow state, they are fully absorbed in the task, so much so that there will be no attentional space left for them to receive information about the self as a separate entity from the task. They will concentrate fully, and they will feel completely in control. There will be no space left in the attention to think about temporal dimensions such as the past or the present as one is fully immersed in the present. The distortion in one’s sense of time means that hours will pass feeling like minutes. Finally, the autotelic experience denotes the fact that flow activities are intrinsically rewarding and are not undertaken for the sake of any external motivators.

In addition to the dimensions described above, three conditions mark the flow state (Csikszentmihalyi, 1975, 1990), i.e., a perceived balance between challenges and skills, clear goals and immediate and unambiguous feedback. With regard to the balance between challenges and skills, flow states occur when the perceived level of the challenge of an activity is slightly above that of one’s skills. If the challenge level is perceived as too high, anxiety will occur, while boredom or apathy is the consequence of low levels of challenge in an activity. However, a challenge level that is perceived to be just above one’s level of skills will trigger a state of flow, encouraging the individual to expand their skill sets to meet the level of challenge. Flow states are dynamic and if one is to experience flow again, they need to heighten their skills to meet a yet higher level of challenge which illustrates the potential of flow theory in explaining individuals’ development over time.

Secondly, clear goals in an activity are paramount to experiencing the flow state. Unambiguous goals will help one to pay attention to relevant stimuli and filter out irrelevant information. However, unclear goals will create disorder in one’s consciousness, preventing them from paying attention to the relevant incoming stimuli (Csikszentmihalyi, 1975, 1990). Finally, immediate and unambiguous feedback plays a critical role in triggering and sustaining the flow state as it enables one to continuously monitor their performance in an activity. In cases where the challenge level is on the rise, skill-building will enable individuals to stay in the flow channel, whereas in circumstances where one’s skill levels exceed the challenge, clear feedback enables one to increase the challenge level so that the flow state will be sustained (Csikszentmihalyi, 1975, 1990).

### Teacher Flow

Research on ESL/English as a Foreign Language (EFL) teachers’ flow state is almost non-existent. In the only study carried out to date, Tardy and Snyder (2004) conducted an exploratory examination of the flow experiences of 10 EFL teachers at a private university in Turkey. Interview data confirmed that the teachers experienced flow inside and outside the classroom. Inside the classroom, flow occurred in moments of high interest and involvement when communication was authentic, and the teachers, as well as the students, felt deep learning was happening. Language teaching has unique attributes which make it different from the teaching of content in several ways (Borg, 2006). On the one hand, the social environment of the EFL classroom gives rise to positive emotions such as enjoyment, pride and happiness and the cognitions that accompany such emotions lead to a heightened focus and concentration, all of which are flow triggering conditions (Dewaele and MacIntyre, 2016). On the other hand, EFL teachers might suffer from language anxiety, high rates of burnout, and threats to their senses of self and identity (MacIntyre et al., 2019) which can impede the flow state. These unique characteristics make it important for more studies to examine the flow state among language teachers (and learners).

In the neighboring fields of psychology and education, a number of studies have been conducted on primary and high school teachers’ flow states. A few studies have compared the frequency and intensity of flow among teachers with those in other professions (Morgan, 2005; Bassi and Fave, 2012). In a study on primary and high school teachers, Bassi and Fave (2012) found that flow proneness was higher among teachers than those holding other jobs, such as physicians, cashiers and white and blue-collar workers. Moreover, the cognitive, affective, motivational and volitional aspects of flow were just as high during classroom teaching as those during hobbies, sports, etc., whereas in other jobs, the motivational and volitional aspects of flow were lower. Other studies have focused on the antecedents of flow, i.e., self-efficacy (Rodríguez-Sánchez et al., 2011) and job resources (Bakker, 2005). Rodríguez-Sánchez et al. (2011) found among a sample of high school teachers that a belief in one’s skills complements the balance of challenges and skills. They proposed a model in which high self-efficacy results in high flow proneness leading to higher levels of the confluence of challenges and skills, which, in turn, predicts a higher frequency of flow over time. Similarly, Bakker’s (2005) study of music teachers showed that resources in the work environment, such as coaching, feedback and social support contribute to a higher balance of challenges and skills, resulting in higher levels of flow among the teachers. Bakker also found support for a form of group flow, i.e., the transfer of flow from teachers to their students, taking over the class group and improving their quality of performance in the classroom. Finally, a study linked teachers’ flow states to their life satisfaction. Olčar et al. (2019) found that the need for competence mediates between intrinsic life goals and flow, leading to higher amounts of life satisfaction among primary school teachers. More specifically, according to Olčar et al.’s (2019) model, intrinsic life goals, such as self-acceptance and contribution to the community (as opposed to extrinsic life goals, such as pursuing financial rewards) lead to higher satisfaction of the need for competence, as a work-related psychological need, resulting in higher amounts of flow at work.

### Flow in Applied Linguistics

Very few empirical studies of flow have been conducted in the field of applied linguistics to date, but there are signs this might change in the years to come. Firstly, the broadening of the field to include holistic concepts and their interrelations has provided more space for situated studies that examine the bigger picture of the phenomena under study (Mercer and Kostoulas, 2018; Dewaele, 2019). In line with this change, researchers have started to explore concepts, such as engagement as a term that carries a broader lens on the learners’ commitment to learning a language, compared to the traditional concept of motivation (e.g., Aubrey et al., 2020; Mercer and Dörnyei, 2020). Flow, as a state of high engagement, provides a possible path down this newly found avenue. Secondly, the recent surge in research on emotions has been so significant that it has been called an emotional turn (White, 2018) or “a sharp increase in interest in emotion” (Dewaele, 2019, p. 2). In this regard, researchers have recently focused their attention on the dynamic interactions of positive and negative emotions, such as enjoyment and anxiety (Dewaele and MacIntyre, 2014, 2016; MacIntyre and Vincze, 2017; Dewaele and Alfawzan, 2018), instead of exclusively exploring negative emotions, e.g., anxiety. Flow theory provides a constructive lens, in this respect, on the interactions between positive and negative emotions (e.g., enjoyment, happiness, anxiety, and boredom). Thirdly, according to Dewaele et al. (2019, p. 5), “the flowering of positive psychology research in applied linguistics” has turned the researchers’ attention to concepts such as empathy, hope, happiness, well-being, optimism, and indeed, flow. As the methodological scope of positive psychology studies expands and more links with existing concepts are made (Dewaele, 2019), the flow state will likely be taken on by a higher number of empirical studies in the future.

Most of the existing studies in applied linguistics examine task flow. Egbert’s (2003) pioneering study sheds light on the nature of language learning tasks that trigger the flow state in the language classroom. She hypothesized that tasks that involved an optimal balance of challenge and skills, high attention and high control, in addition to high interest generate flow for the learners in the classroom. To test this hypothesis, she assigned seven communicative tasks to fourth-semester university students learning Spanish as a foreign language. Her results indicated that the tasks that scored higher along the above-mentioned dimensions generated higher degrees of flow in the classroom. The highest flow-triggering task was one that involved open-ended electronic chatting with native speakers of Spanish, which caused high flow due to its interactive nature. Another task, which included choosing a mystery persona and having the participant’s classmates guess who it was via email, also created intense flow due to the choice and autonomy it provided. Based on these results, Egbert concluded that teachers are capable of designing tasks that create flow in the English language classroom environment. Furthermore, in a study on EFL learners, Cho (2018) found that writing tasks that included interactive speaking parts created a higher degree of flow than tasks that focused exclusively on speaking. As a result, Cho (2018) concluded that task modality had a significant effect on the balance of challenge and skills and consequently on flow.

Czimmermann and Piniel (2016) investigated classroom flow, task-specific flow and anti-flow experiences among advanced Hungarian university-level language learners. Their results indicated that the majority of their participants experienced both classroom and task-specific flow. Most participants experienced above-average amounts of classroom flow in their English classes, and average flow with a picture Domino task conducted in the study. As expected, the anti-flow experiences, i.e., those that impeded the flow experience, were identified as boredom, apathy, and anxiety. Finally, Aubrey (2017) compared the amount of flow Japanese learners in an inter-cultural group experienced as opposed to the flow experienced in an intra-cultural group. He found that tasks that provided opportunities for intercultural communication resulted in more cognitive and emotional engagement, and consequently, triggered more flow than those that only included contact between Japanese students. He posited that intercultural communication led to more turn-taking and interactivity which brought about more positive experiences for the students.

## Emotional Intelligence, the Big Five and Flow

Emotional intelligence is defined as: “the ability to monitor one’s own and others’ feelings, to discriminate among them, and to use this information to guide one’s thinking and action” (Salovey and Mayer, 1990, p. 189). Arguably, entering and sustaining the flow state is an emotional process which needs to be effectively managed (Marin and Bhattacharya, 2013; Freire and Tavares, 2016; Tavares and Freire, 2016; Xie, 2021). According to Csikszentmihalyi (1990), navigating the small space between two negative emotions, i.e., boredom and anxiety, results in entering the flow channel. Once the activity is over, a positive emotion, namely, enjoyment, is experienced which compels one to experience flow on future occasions. Moreover, empirical studies have associated flow proneness with low impulsiveness and stable emotions (Ullén et al., 2012; Ross and Keiser, 2014). In this regard, individuals who use effective emotional regulation strategies will experience flow more often. Goleman (1995, pp. 180–181) hypothesizes:

Being able to enter flow is emotional intelligence at its best; flow represents perhaps the ultimate in harnessing the emotions in the service of performance and learning. In flow, the emotions are not just contained and channeled, but positive, energized and aligned with the task at hand.

However, despite the fact that the connection between emotions and flow is self-explanatory (as stated above), empirical studies investigating the relationship between emotional intelligence and flow are scarce. In applied linguistics, emotional intelligence has been explored with regard to learners’ and teachers’ experiences inside and outside the classroom (Gregersen et al., 2014), teachers’ self-reported creativity, pedagogical skills and classroom management (Dewaele et al., 2018), and learners’ foreign language enjoyment and anxiety (Li and Xu, 2019), but no study has examined the relationship between emotional intelligence and flow to date. In the field of musical performance, a study (Marin and Bhattacharya, 2013) found that flow was predicted by the amount of daily practice and emotional intelligence. Other factors, such as age, gender, age of first piano lessons and years of musical training were found insignificant.

With regard to personality traits, five broad, overarching traits have been found to subsume other lower-order traits and characteristics (McCrae and Costa, 2008). These traits include conscientiousness, openness to experience, agreeableness, neuroticism and extraversion. Each of these traits has, more or less, been studied regarding different aspects of second language learning. For example, extraversion has been examined in relation to oral fluency (Ockey, 2011) and speaking anxiety (Hamedi et al., 2015), openness to experience has been studied with respect to creativity (Chen et al., 2019) and self-perceived English L2 proficiency (Ożańska-Ponikwia and Dewaele, 2012) and agreeableness has been explored in connection with non-verbal communication (Sitra and Abdullah, 2017) and peer-assessment (Birjandi and Siyyari, 2016). Moreover, links between conscientiousness and intended effort (Ghorbani and Rashvand Semiyari, 2020) and neuroticism and foreign language anxiety (Dewaele, 2013) have been made. However, yet again, flow has not been studied in relation to the big five in applied linguistics.

In the field of psychology, a few studies have examined the relationship between flow proneness and the big five factors (Ullén et al., 2012; Bassi et al., 2014; Ross and Keiser, 2014). In this regard, these studies have found a positive correlation between conscientiousness and flow proneness and a negative correlation between neuroticism and the propensity to be in flow (Ullén et al., 2012; Ross and Keiser, 2014). Given the fact that conscientious individuals are focused and active, have self-discipline and show persistence in the pursuit of their goals (McCrae and Costa, 2008), they are able to keep their attention on relevant features of the task at hand and sustain the flow state. Regarding neuroticism, it can be hypothesized that the emotional and cognitive instability resulting from this trait diverts attention from the task and prevents one from experiencing positive emotion, i.e., enjoyment, which is a necessary condition for entering and sustaining flow.

However, findings regarding the rest of the big five measures (i.e., apart from conscientiousness and neuroticism) are somewhat mixed (Nakamura et al., 2019). Extraversion, especially facets such as assertiveness, activity, and cheerfulness are associated with general interest and enjoyment in life. Therefore, some studies have found a positive relationship between extraversion and flow proneness (Ross and Keiser, 2014). With regard to openness to experience, the broad interest and curiosity in life are related to autotelic behavior, but according to Tse et al. (2020a), most flow measurements do not aim to examine these two elements, which explains why a positive relationship between this trait and flow proneness has not often been reported in the literature. Finally, with respect to agreeableness, an insignificant relationship between this trait and flow proneness or autotelic behavior has been reported in most studies (Ullén et al., 2012; Bassi et al., 2014). Tse et al. (2020a) posit that since the autotelic personality is not associated with the social environment, facets such as altruism and sympathy, which are socially focused, might not play a significant role in autotelic behavior. Therefore, a weak relationship between this trait and flow proneness is hypothesized.

## Research Questions

Taking into account the importance of teachers’ flow states in creating optimal classroom environments and enhancing the growth and well-being of their students, the uniqueness of teaching EFL compared to other subjects and the fact that more research investigating the connections between flow, the Big Five and emotional intelligence is needed, this study seeks to answer the following research questions:

(1) What is the quality of experience of “a purposeful sample of Iranian” EFL teachers inside and outside the classroom?
(2) What is the relationship between the EFL teachers’ flow state and their Big Five personality traits?
(3) What is the relationship between the EFL teachers’ flow state and their emotional intelligence?

## Materials and Methods

### Participants

Seventy-five EFL teachers teaching adult classes in the city of Mashhad, in Northeastern Iran participated in this study. The participant sample comprised of 54 female and 21 male teachers (one male participant withdrew from the study). The teachers taught in four language schools and testing centers across the city and had higher-intermediate to advanced language proficiency as measured by the proficiency tests they had taken in their teaching institutions. All of the teachers were L1 speakers of Persian/Farsi. As communicative language teaching/learning is not the primary objective of higher educational institutions in Iran, a sizable population of young adults attend private language schools and testing centers to learn conversational English or prepare for international exams such as IELTS and TOEFL. The teachers who participated in this study taught in four popular schools within this context. They were mostly undertaking their higher education studies in Iranian universities and were teaching part-time in the language schools. All of the participants were in their twenties or thirties.

At the outset of the study, a 90-minute informational session was arranged with the teachers, where the researcher explained the main objectives of the study and provided training on the concepts included in the questionnaires and the ways of filling them out. All 75 teachers gave written consent to (a) participating in this study, (b) filling out the questionnaires, and (c) receiving multiple text messages a day as per the design of the study. Two of the questionnaires were completed in the language schools and one was filled out multiple times at home.

### Instruments

#### Experience Sampling Forms

In this study, flow was measured through the multi-time Experience Sampling Method (ESM; Hektner et al., 2007). The ESM is used to examine the participants’ quality of experience at different times of the day. The participants typically receive around 8 beeps/messages on an electronic device per day for a period of 7 days and are asked to fill out an ESF no longer than 20 minutes after receiving each beep/message.

The ESF used in this study consisted of 38 items, 30 of which could be numerically coded and consisted of Likert-type scale items on affect (e.g., happy, proud, cheerful, and sociable), activation (alert, active, strong, and excited), cognitive efficiency (concentration, ease of concentration, self-consciousness, and clear mood), and motivation (wish to do the activity, control, feeling involved) (Csikszentmihalyi, 2014, p. 38). Moreover, the ESF provided open-ended questions that elicited information about the time and location of measurement, general mood and information about the activity/activities conducted at the time of measurement. The reliability and validity of the ESF has been demonstrated by Hektner et al. (2007) and Csikszentmihalyi (2014) with the internal consistency measure (Cronbach’s alpha) in the acceptable range (between 0.70 and 0.94) across the different studies. With regard to the construct validity of the open-ended sections of the ESF, both Csikszentmihalyi and Graef (1980) and Robinson (1985) [as cited in Hektner et al. (2007)], reported rank-order correlations of above 0.90 between the ESF and similarly coded diary-based studies of the participants’ quality of experience in different activities (for more information about the validity of the ESF, see Hektner et al., 2007).

#### The Emotional Quotient Inventory

In this study, a Persian adaptation, which is a shorter 90-item version of BAR-On EQ-i (Bar-On, 1997, 2004), was used to measure emotional intelligence. The reliability of this questionnaire has been measured by Samouee (2002) with Cronbach’s Alpha reported as high as 0.95. The EQ-i has five scales and several subscales, namely, Intrapersonal EQ (self-regard, emotional self-awareness, assertiveness, independence, and self-actualization), Interpersonal EQ (empathy, social responsibility, and interpersonal relationships), Stress management EQ (stress tolerance and impulse control), Adaptability EQ (reality testing, flexibility, and problem- solving), and General Mood EQ (optimism and happiness). Each item has a five-point Likert-type scale ranging from “1-very seldom or not true of me” to “5-very true of me or true of me.”

#### The Revised NEO Personality Inventory

A Persian adaptation of the 60-item NEO Personality Inventory was used in this study. The reliability of this questionnaire has been measured by Garousi et al. (2001) with Cronbach’s Alpha ranging between 0.66 and 0.87 for the different scales. The original version of NEO-PI-R has 240 items and uses a five-point scale (just like the shorter version). The internal consistency coefficient is between 0.85 and 0.95 with evidence for construct, convergent and divergent validity being provided by Costa and McCrae (2008). The main facets and sub-facets for both the shorter and original measures include conscientiousness (competence, order, dutifulness, achievement striving, self-discipline, and deliberation), neuroticism (anxiety, hostility, depression, self-consciousness, impulsiveness, and vulnerability), extraversion (warmth, gregariousness, assertiveness, activity, excitement-seeking, and positive emotions), openness to experience (fantasy, aesthetics, feelings, actions, ideas, and values), and agreeableness (trust, straightforwardness, altruism, modesty, and tender-mindedness). Each item includes a five-point Likert-type scale ranging from 1-strongly disagree to 5-strongly agree.

### Procedure

As per the ESM (Hektner et al., 2007), every participant completed 8 ESFs per day, after receiving 8 text messages at random times between 9:00 am and 9:00 pm. Upon receiving a text message, the participants had 20 minutes to fill out an ESF, with ESFs completed after this time period discarded. It was also arranged that no two text messages would be sent sooner than 30 minutes from each other. As filling out the ESFs was a recurring activity, the participants reported that each form did not take more than 3 minutes to complete once they had become familiar with its format and structure. The process of ESF completion took 7 days with each participant filling out a total of 56 ESFs.

At the end of the week, the researcher collected the ESFs and administered an EQ-i and a revised NEO Personality inventory. The former took approximately 30 minutes while the latter took 40 minutes to complete. To ensure confidentiality and anonymity, each participant had been assigned a code that they wrote on their ESFs, EQ-i, and NEO Personality questionnaires.

### Data Analysis

More than 4,000 ESFs were coded numerically, and the data were entered on SPSS 19. As per the previous studies (Egbert, 2003), responses higher than 70% of the total absolute score (184 in this study) were interpreted as flow. Subsequently, the data from the EQ-i and the Revised NEO Personality Inventory were coded and entered on SPSS.

Correlations and independent samples *t*-tests were run to investigate the relationships between flow and EQ and flow and the Big Five. For the *t*-tests, the teachers were divided into the high flow (autotelic) and low flow (non-autotelic) groups based on their flow scores throughout the sampling week. The *t*-tests indicated the difference between the means of the autotelic and non-autotelic groups for the EQ-i scales and the Big Five personality traits. To find out the best predictors of the flow state, multiple regression analysis was run.

The open-ended sections of the ESFs increased the reliability of the quantitative section by providing qualitative data about the kinds of activities the participants were engaged in at the time of receiving the text message. Data about these activities were coded by hand by the researcher. The emerging categories were identified, and percentages based on the frequency of occurrence of each activity in the data were calculated.

## Results

### Teachers’ Quality of Experience

**FIGURE 1:**
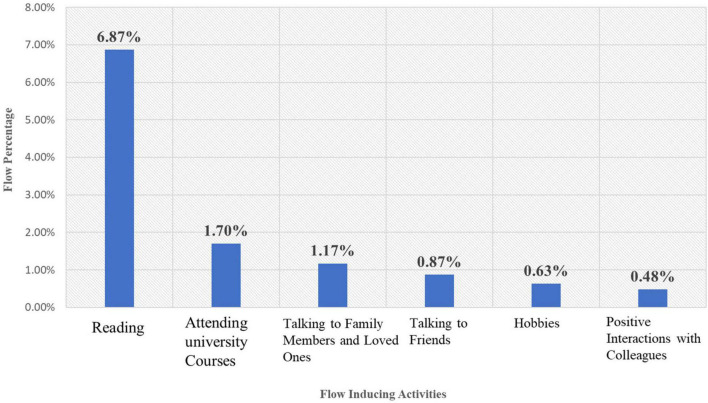
Teachers’ quality of experience outside the classroom.

**FIGURE 2:**
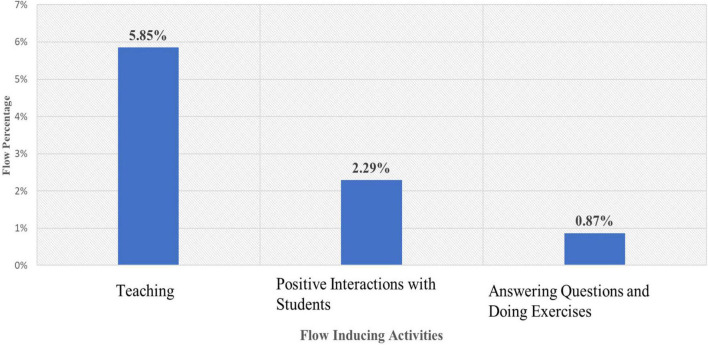
Teachers’ quality of experience inside the classroom.

Overall, around 21% of the ESFs completed indicated that the participants were in the flow state. Approximately, 12% of the ESFs revealed that the flow state had occurred outside the classroom and 9% signaled the occurrence of flow inside the classroom. The most prominent flow triggering activity outside the classroom was reading textbooks (see Figure 1). Inside the classroom, teaching and delivering lessons was the most frequently reported flow-triggering activity (see Figure 2).

Other activities such as attending university classes, talking to family members and loved ones and pursuing hobbies (happening outside the classroom), and having positive interactions and answering questions and doing exercises (occurring inside the classroom) were less often reported as flow triggering by the participants in the sample.

With regard to the extensiveness of the two most frequently reported flow-inducing activities across the sample, 85% of the participants reported being in flow at least once while reading outside the classroom and 87% reported being in flow at least once while teaching during the sampling week.

### Correlational Analysis: The Relationship Between Flow and the Big Five

With regard to correlations between the Big Five and the teachers’ flow state, it can be seen that all Big Five factors except agreeableness had a statistically significant relationship with flow (see Table 1). Conscientiousness had a moderate correlation (*r* = 0.428, *p* = 0.00) and neuroticism had a significant reverse relationship with flow (*r* = −0.360, *p* = 0.00). As seen, the correlation between agreeableness and flow was statistically *insignificant* (*r* = 0.200, *p* = 0.087).

**TABLE 1:** Correlations between the teachers’ flow state and the big five factors.

	Neuroticism	Extraversion	Openness to experience	Agreeableness	Conscientiousness
Flow	–0.360	0.384	0.372	0.200	0.428
Sig.	0.002	0.001	0.001	0.087	0.000

### Correlational Analysis: The Relationship Between Flow and Emotional Quotient Inventory

As it could be seen in Table 2, the correlations between the teachers’ flow state and total EQ as well as all other EQ-i subscales were statistically significant. The strongest correlation was in the case of total EQ (*r* = 0.397, *p* < 0.0001), and the weakest was in the case of intrapersonal (*r* = 0.299, *p* = 0.10).

**TABLE 2:** Correlations between the teachers’ flow state and five main EQ-i subscales.

	Total EQ	Intrapersonal	Interpersonal	Stress management	Adaptability	General mood
Flow	0.397	0.299	0.336	0.384	0.324	0.349
Sig.	0.000	0.010	0.003	0.001	0.005	0.002

### *T*-Test Results: High and Low Flow Groups and the Big Five Personality Traits

The results of the independent samples *t*-test indicated that the difference between the means of high flow (autotelic) and low flow (non-autotelic) groups was significant for all personality traits except for agreeableness (see Table 3).

**TABLE 3:** The difference between the means of high flow (autotelic) and low flow (non-autotelic) teachers for the big five personality measures.

Flow	High and low flow groups	*N*	Mean	SD	*T*	Df	Sig.
Neuroticism	High	32	22.12	7.76	2.88	72	0.005
	Low	42					
Extraversion	High	32	27.56	6.1	−2.02	72	0.046
	Low	42					
Openness to experience	High	32	28.75	4.28	−2.21	72	0.03
	Low	42					
Agreeableness	High	32	31.71	6.76	−0.81	72	0.418
	Low	42					
Conscientiousness	High	32	30.03	8.65	−3.15	71	0.002
	Low	42					

### *T*-Test Results: High and Low Flow Groups and Emotional Quotient Measures

The independent samples *t*-test was run to calculate the difference between the means of high flow (autotelic) and low flow (non-autotelic) groups for each EQ-i subscale. As it could be seen in Table 4, the difference between the means of the two groups was significant for total EQ and all other EQ-i subscales.

**TABLE 4:** The difference between the means of high flow (autotelic) and low flow (non-autotelic) teachers for EQ-i subscales.

Flow	High and low flow groups	*N*	Mean	SD	*T*	Df	Sig.
Total EQ	High	32	327.65	40.29	−2.75	72	0.008
	Low	42					
Intrapersonal	High	32	110.66	17	−2.88	72	0.005
	Low	42					
Interpersonal	High	32	70.75	13.19	−2.33	72	0.022
	Low	42					
Stress	High	32	33.4	11.46	−2.198	72	0.032
	Low	42					
Adaptability	High	32	61.31	8.49	−2.66	72	0.01
	Low	42					
Mood	High	32	45.5	8.01	−2.28	72	0.025
	Low	42					

### Multiple Regression Analysis: The Best Predictors of the Flow State

**FIGURE 3:**
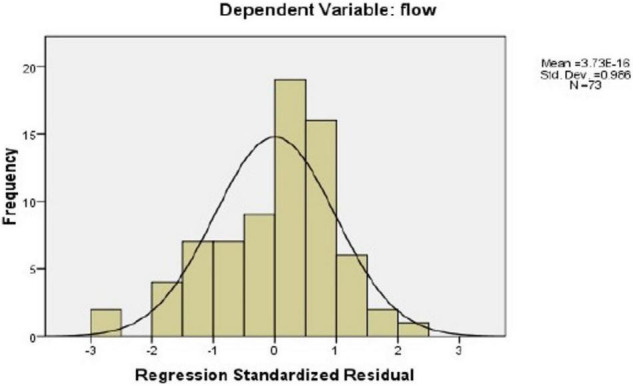
Histogram of the residuals.

**FIGURE 4:**
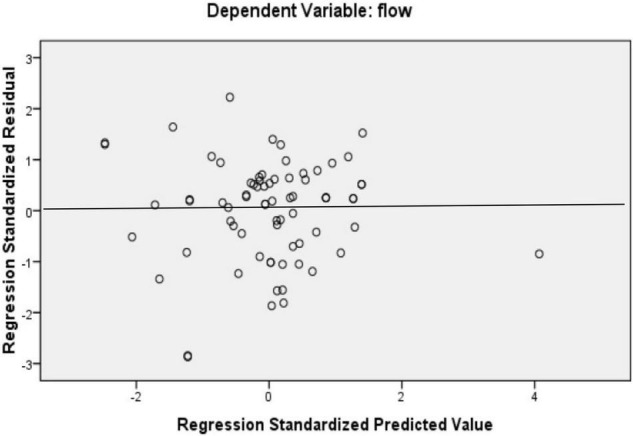
The scatter plot of the variance.

To find out the best predictors of the flow state, multiple regression analysis was run (see Table 5). Figures 3, 4 show that the variances were equally distributed, and therefore, the conditions for running a regression analysis were met. More specifically, Figure 3 shows that the regression residuals were normally distributed, and Figure 4 indicates that the variance of the residuals did not vary much. Based on the *R*^2^ values, *conscientiousness* and *conscientiousness and openness to experience* were the best predictors of the flow state. It can be stated that teachers’ scores on conscientiousness predicted 22% of the variance in flow scores and their scores on conscientiousness and openness to experience predicted 33% of the variance in flow scores. The effect size for conscientiousness is medium, *f*^2^ = 0.28, and for conscientiousness and openness to experience is large, *f*^2^ = 0.49.

**TABLE 5:** The best predictors of the flow state.

Predictors	*R*	*R* ^2^	Adjusted *R*^2^	*F*	β	*f* ^2^	Sig.
Conscientiousness	0.469	0.220	0.209	20.016	0.469	0.282	0.000
Conscientiousness, openness to experience	0.577	0.332	0.313	17.434	0.374	0.492	0.000

## Discussion

This study examined a sample of 75 EFL teachers’ quality of experience in flow and the existing relationships between their flow experiences, their emotional intelligence and their Big Five personality traits. With regard to the first research question, it was found that the teachers’ quality of experience did not involve too much variation and complexity within the week they were sampled. Outside the classroom, “reading textbooks” (with the aim of preparing for university courses), and inside the classroom, “teaching and delivering lessons” were the two most frequently reported flow-triggering activities. Hobbies and interacting with their family members, loved ones, friends and colleagues did not seem to make a significant difference in their recurring flow experiences. The teachers’ age range and their educational status might help shed light on this. They were in their twenties or thirties and were teaching part-time while pursuing their higher education studies on a full-time basis (at the time this study was conducted). Therefore, the two main professional activities in their lives, i.e., studying and part-time teaching seem to have triggered the majority of their flow experiences whereas other activities did not seem to create too much flow. Moreover, the percentages of the teachers’ flow experiences inside and outside of the classroom, especially the percentages of their main flow triggers (i.e., reading and teaching, see Figures 1, 2), were quite close. This finding is in line with studies that report teachers being able to find equal opportunities to experience flow inside and outside the classroom -as opposed to professions in which the participants experienced a significantly higher amount of flow outside of their work context (e.g., physicians, cashiers and white and blue-collar workers, Bassi and Fave, 2012).

The fact that language teachers were able to create similar opportunities to experience flow inside and outside the classroom is a significant finding due to the uniqueness of language teaching compared to the teaching of content (Borg, 2006). The EFL classroom is characterized by a high frequency of positive and negative emotional experiences, identity changes, motivational fluctuations and the teaching of personally and culturally meaningful content (MacIntyre et al., 2019). It seems that in this study, EFL teachers were able to leverage the flow-triggering conditions offered by their profession and remain less scathed by issues such as the precariousness of their teaching positions, burnout, or language anxiety (Horwitz, 2017). This is important given the benefits of the flow state for the EFL teachers and their students. Inside the classroom, flow can cross over from teachers to students (and vice versa) (Bakker, 2005), creating an effective learning environment characterized by intrinsic motivation (Hektner and Csikszentmihalyi, 1996), academic achievement (Joo et al., 2015), and high self-esteem (Mao et al., 2020). Outside the classroom, teachers’ flow states can improve their well-being (Tse et al., 2020b). As research on this construct is gathering interest in applied linguistics (especially in the times of the COVID-19 pandemic) (MacIntyre et al., 2020; Mercer and Gregersen, 2020), flow can be regarded as a coping strategy for dealing with stress, regulating emotions and ultimately achieving improved mental and emotional well-being (Tse et al., 2020b).

With regard to the second research question, multiple regression analysis revealed that conscientiousness and openness to experience were the best predictors of the teachers’ flow state and together predicted 33% of the variance in flow scores- with the effect size calculated as large for the combination of these two traits. This is a substantive finding as it, firstly, confirms the results of the previous studies reporting conscientiousness as the main personality trait correlating with flow (Ullén et al., 2012; Ross and Keiser, 2014; Tse et al., 2020a). The reason for this might be that this trait includes sub-facets such as self-discipline, organization, goal-orientation and attentional control that are fundamentally flow-inducing (Csikszentmihalyi, 1990). However, in addition to conscientiousness, this study reveals that personality characteristics such as imagination, curiosity, creativity, and originality (associated with openness to experience) might also be important in increasing the teachers’ frequency and intensity of flow. Taken together, these findings suggest that teachers who can set clear instructional goals, can focus their attention on the relevant features of teaching activities, and have self-control and also have a strong imagination, are inventive in the classroom and do not shy away from trying new activities might have a higher degree of flow proneness.

Moreover, Egbert’s (2003) study indicated that tasks that involve a high balance of challenge and skills, require high attention, give learners a high sense of control and are interesting and novel, trigger more flow. Although this study did not directly explore teachers’ usage of learning tasks, the flow-inducing personality traits found here (i.e., conscientiousness and openness to experience) might support the task characteristics mentioned above. That is, teachers who are conscientious can tolerate higher levels of task challenge and will engage in the building of their skills to meet those heightened levels of challenges (Ullén et al., 2012; Ross and Keiser, 2014). They are also better equipped to direct their attention and stay in control of the task at hand (Tse et al., 2020a). By the same token, teachers who are more open to experience tend to search for novelty and activities that can excite their curiosity and imagination (McCrae and Costa, 2008). Therefore, it can be hypothesized that teachers with a high level of conscientiousness and openness to experience will likely experience flow when engaged in tasks that include the characteristics mentioned above (i.e., high challenge and skills, high control and high interest and novelty) and will also actively create classroom tasks that involve these flow-inducing characteristics for their learners.

In this study, agreeableness was not found to be correlated with the teachers’ flow state. As mentioned, teachers’ flow was mainly triggered by activities such as reading or classroom teaching. Interacting with loves ones, family members, friends and colleagues did not seem to be major flow contributors, which might explain the lack of relationship found between agreeableness and flow. That is, many of the facets of agreeableness such as trust, altruism and empathy are socially focused which did not seem to generate much flow in this study.

Regarding the third research question, this study found evidence for the significance of the correlations between flow and all of the EQ-i facets. Also, independent samples *t*-test indicated a group-wise significant difference between the mean scores of autotelic and non-autotelic teachers for all of the EQ-i facets. These findings support the emphasis placed upon emotional awareness and emotional regulation for entering and sustaining the flow state (Marin and Bhattacharya, 2013; Freire and Tavares, 2016; Tavares and Freire, 2016; Xie, 2021). Therefore, the extent to which teachers can regulate their mood, control their stress, adapt to different classroom situations and understand and manage their own as well as others’ emotions (including those of their students’), seems to determine how frequently they will be in the flow state. Drawing on Fredrickson’s broaden and build theory of positive emotion (2004), MacIntyre and Gregersen (2012) posit that positive emotions broaden individuals’ attention and increase their approach motivation, while negative emotions narrow their focus and cause them to withdraw from the task at hand. Taken together, these findings suggest that teachers with high EQ-i scores might leverage their positive emotions and regulate their negative emotions to enter and remain in the flow channel more often than others inside and outside of the classroom.

## Conclusion

The purpose of this study was to analyze EFL teachers’ experiences in flow and to explore the relationships between their emotional intelligence, personality traits and the flow state. The data show that teachers were able to create similar opportunities for experiencing flow inside and outside the classroom. This finding indicates that teaching was as flow-inducing for this sample of language teachers as their main outside-of-the-classroom activity, i.e., reading for university courses. Such recurring flow states have several benefits for teachers (and their students) both inside and outside the classroom, and it is high time that future studies explore flow proneness in other language teaching contexts to increase the understanding of the field of the nature of flow-triggering activities undertaken by language teachers around the world.

Secondly, two Big Five personality traits, namely, conscientiousness and openness to experience were the strongest predictors of the flow state. This shows that teachers with greater self-discipline, attentional focus and goal orientation and stronger imagination, curiosity and inquisitiveness might experience more flow and also create the conditions for their students to be in the flow channel more frequently than teachers who do not possess these qualities. Finally, it was found that all emotional intelligence facets correlated significantly with the flow state which highlights the importance of teachers’ emotional stability and emotional regulation for entering and sustaining the flow state. Overall, the more the conditions for experiencing teacher flow are explored and the teachers’ inner psychological states, as well as task characteristics and contextual elements are examined, the higher the possibility that the flow state will be recreated in the language classroom, leading to increased opportunities for the growth, engagement and well-being of learners and their teachers.

### Implications for Language Teaching

This study has several implications for language teaching. Firstly, it is important for language teachers to continually increase their knowledge of flow theory and seek to recreate the flow state in the classroom given the benefits of the flow state for themselves and their students. One way they can achieve this is through the administration of tasks that challenge (but do not overwhelm) their learners (Csikszentmihalyi, 1975, 1990). Through timely and sensitive feedback, teachers can help their learners adjust the challenge of the task with their current level of skills and remain in control of the activity. Tasks might also encourage deep learning, curiosity, and imagination (Nakamura and Csikszentmihalyi, 2002) among language learners. This study did not directly investigate the usage of tasks by language teachers. However, teachers who possess the personality traits found to be associated with the flow state (i.e., conscientiousness and openness to experience) might encourage the above-mentioned task characteristics in the classroom. That is, teachers with high levels of conscientiousness might model the adjusting of challenges and skills for their learners and those with a high openness to experience might introduce tasks that encourage imagination, curiosity and originality to their learners. Teacher training courses will do well to emphasize these qualities as teacher candidates and novice teachers practice designing classroom tasks that highlight the characteristics mentioned above, with the aim of enhancing the flow experience within the ESL/EFL classroom environment.

Moreover, although personality traits have traditionally been treated as relatively stable constructs in psychology (Costa and McCrae, 1986), recent studies have shown that they are dynamic and adaptive (despite exhibiting stability at their core) (Dörnyei and Ryan, 2015). Research has also highlighted the role of life experiences as important elements which can lead to behavioral change throughout the course of one’s life (Roberts and Mroczek, 2008; Kawamoto, 2016). Viewed in this light, language teachers might benefit from learning to be more conscientious and open to new experiences to more frequently experience the flow state. In other words, practicing organizational skills, self-discipline and attentional focus along with inquisitiveness (i.e., trying out new activities) and thinking imaginatively might help language teachers to be in the flow channel more often. Teacher training that involves the practice of metacognitive strategies such as mental contrasting (i.e., envisioning one’s goals and the obstacles that can appear in one’s path), if-then scenarios and episodic future thinking (i.e., visualizing detailed episodes of future events) might be helpful in this respect (see Javaras et al., 2019).

Finally, in this study, emotional intelligence was found to be correlated with the flow state. Therefore, it might be helpful for EFL teachers to practice emotional regulation and stress management strategies to enable themselves (and their learners) to more easily and frequently experience flow. In this regard, mindfulness might help teachers regulate their emotions and enhance their well-being (Mercer and Gregersen, 2020). Moreover, teacher training courses might guide teacher trainees on ways of managing the emotional environment of the classroom so that it is more conducive to experiences of flow. The more the teachers manage their own as well as their learners’ negative emotions and the more they capitalize on positive emotions, the deeper the students’ learning will be and the more the flow state will become a common practice within the classroom environment.

### Limitations and Suggestions for Future Research

This study has a few limitations. Firstly, personality was measured based on McCrae and Costa’s (2008) Big Five Factor Model. Adopting other views of personality (e.g., the New Big Five- McAdams and Pals, 2006) and other trait models (e.g., Myers-Briggs Type Indicator, Myers, 1978) can provide a more thorough understanding of the relationships between personality and the flow state. Moreover, future studies might benefit from a bigger sample size. In this study, despite the small sample size, multi-time experience sampling was used (i.e., a total of 56 ESFs completed by each participant), but if practical, studies might utilize both a larger sample size and the experience sampling method in the future. Finally, the teachers’ quality of experience (e.g., the flow-inducing activities) is context-specific in this study. The findings of future studies regarding language teachers’ quality of experience in other teaching contexts around the world can complement the findings of this study and, therefore, be of benefit to the field of applied linguistics.

Moreover, it is commonly assumed that when learners are in flow, language learning is happening, but that assumption needs to be tested more extensively (Egbert, 2003). One direction for future studies, as Egbert (2003) points out, is to examine the connections between flow and “language learning outcomes” (p. 514). In this regard, Egbert’s study revealed anecdotal evidence for the relationship between flow and noticing, but more research is needed to explore the connections between flow and language skills, language systems and other broader competencies. Moreover, the role of language teachers in planning for and creating classroom flow (e.g., by designing tasks and managing the classroom environment) also needs to be studied. With regard to task flow, it would be interesting to explore different task characteristics or dimensions to see which are potentially more flow-inducing (Egbert, 2003).

It was suggested that teachers practice conscientiousness and openness to experience in order to enhance the frequency and intensity of their flow experiences. To this aim, the usage of metacognitive strategies such as mental contrasting, if-then scenarios and episodic future thinking were recommended. A direction for future studies to take in this respect would be to explore whether teachers (especially non-autotelic personalities) can enhance their flow proneness by utilizing the above-mentioned strategies. As such, it would be interesting to see whether the introduction of these strategies in teacher workshops could increase teachers’ flow scores especially those who consistently score lower in their ESFs (i.e., non-autotelic teachers).

Finally, it is recommended that future studies consider the whole picture when it comes to investigating language teachers’ flow state. If the aim is to study teachers’ behavioral patterns, it might be a good idea to explore the interactions of their personality traits and the characteristics of the classroom tasks they assign as well as the qualities of the learning context that trigger teacher and learners’ flow states. Clarifying the nuances of these relationships will help the flow state be better understood and recreated within language learning classrooms in the future.

## Data Availability Statement

The original contributions presented in the study are included in the article/supplementary material, further inquiries can be directed to the corresponding author.

## Ethics Statement

Ethical review and approval was not required for the study on human participants in accordance with the local legislation and institutional requirements. Participants provided written informed consent to participate in the study.

## Author Contributions

The author confirms being the sole contributor of this work and has approved it for publication.

## Conflict of Interest

The author declares that the research was conducted in the absence of any commercial or financial relationships that could be construed as a potential conflict of interest.

## Publisher’s Note

All claims expressed in this article are solely those of the authors and do not necessarily represent those of their affiliated organizations, or those of the publisher, the editors and the reviewers. Any product that may be evaluated in this article, or claim that may be made by its manufacturer, is not guaranteed or endorsed by the publisher.
